# Effect of quince seed gum (QSG) on the performance of injectable hyaluronic acid hydrogels in terms of the rheological, morphological, and mechanical aspect

**DOI:** 10.55730/1300-0527.3669

**Published:** 2024-05-29

**Authors:** Serdar KOLAY, Nilhan KAYAMAN APOHAN, Erdinç BABUÇ, Gökay GÜN

**Affiliations:** 1 Department of Chemistry, Institute of Pure and Applied Sciences, Marmara University, İstanbul, Turkiye; 2 World Medicine, İstanbul, Turkiye; 3 Department of Chemistry, Faculty of Science, Marmara University, İstanbul, Turkiye

**Keywords:** Hydrogel, quince seed gum, rheology, dermal filler injection, intraarticular injection

## Abstract

Injectable hydrogels play an important role in tissue engineering as a filling and repairing material. This study aimed to develop a new injectable hydrogel based on hyaluronic acid (HA) and quince seed gum (QSG) and investigate the effect of QSG on hydrogel performance. The amount of unreacted 1,4-Butanediol diglycidyl ether is maintained at an undetectable level for HA-QSG hydrogels. Amino acid analysis showed that the HA-QSG hydrogel had rich amino acid concentrations of leucine, arginine, and valine. After thermal sterilization, the elastic modulus of HA-QSG gels for dermal and intraarticular filler applications is 63 Pa and 92 Pa, respectively. Pore size was found below 200 μm and the dense homogeneous pore structure was observed.

## 1. Introduction

Hydrogels are hydrophilic biomaterials that can absorb large amounts of water or biological fluids and they are excellent candidates for biomedical applications in drug delivery and tissue engineering [1–3]. The swelling of hydrogels in water is mainly due to the short-range interactions of ionic groups, such as carboxylic acid, amide, hydroxyl, sulfonic acid, and amino groups [4,5]. The water-holding properties of hydrogels can vary depending on their pore size and crosslink density [6]. Hydrogels can be synthesized from natural polymers, polysaccharides, and proteins to synthesize biocompatible materials with low cytotoxicity for cell regeneration and tissue engineering [7]. Hyaluronic acid (HA) is a natural polymer and consists of D-glucuronic acid and N-acetylglucosamine groups linked together by repetitive β-1,4 and β-1,3 glycosidic bonds [8]. The molecular weight of HA can vary as a single-chain polymer from 10^5^ to 10^7^ kDa [9]. Hyaluronic acid is a widely used material in tissue scaffolding and cosmetic fields and plays an important role in processes such as wound healing and cell signaling [10–12]. Vertebrate organisms and bacteria naturally produce hyaluronic acid. In the human body, HA is mostly found in the extracellular matrix of connective tissues, synovial fluid of joints, the dermis of the skin, and around the eyes [13].

HA, which plays an important role in modern medicine and drug releases, is becoming widespread as an injectable medical device in tissue engineering and cosmetic surgery [14–16]. In many medical applications involving the human body, HA is not preferred due to the weak mechanical properties of its natural straight-chain structure and its rapid in vivo degradation [17–19]. Hyaluronidase catalyzes the rapid degradation of linear HA in the skin and has a half-life of 2–3 days [20]. In many studies, chemical cross-linking methods have been preferred by utilizing the functional groups (-OH, -NHCOCHCH_3_, -COOH) of polymer to prevent the easy disintegration of hyaluronic acid. Additionally, because HA is cross-linked with a small amount of cross-linker, it retains many of its hydroxyl groups after cross-linking, preserving its water absorption properties. This characteristic is crucial for its use as a filling material [21,22].

The reaction between crosslinking agent 1,4-Butanediol diglycidyl ether and linear HA is the most preferred crosslinking method [23]. The cross-linking process increases the mechanical and in vivo properties of HA by converting it from a straight chain to a network structure [24]. HA can be blended with natural and synthetic polymer mixtures to improve its properties. Combining HA with polysaccharides is one of the best approaches to reducing costs. In a recent study, the blends of hyaluronic acid with collagen and chitosan were reported [25–27].

Quince is a native fruit of the West Asian region and belongs to the family Rosaceae [28,29]. The mucilage in the seeds can be extracted by soaking in water, producing what is known as quince mucilage (QCM) [30,31]. QCM is a plant biopolymer with a polysaccharide-protein structure containing flavonoids, sterols, alkaloids, tannins, saponins, resin, phenol, and terpenoids [32,33]. QCM contains a greater ratio of glucuronic acid residues (carboxylic acid), which makes it a slightly acidic hydrogel. Therefore, in the alkaline buffer of pH 6.8–7.4, it shows higher swelling [34]. The freeze-drying process is crucial to prevent undesirable changes in the physical and chemical properties of the dried quince seed mucilage (QSM). The amino acids in freeze-dried seeds were identified, and it was found that L-aspartic acid, L-glutamic acid, and L-aspargine constitute about 60%–75% of total amino acids [35]. QSM is medically used for the treatment of asthma and for relieving cough and chest discomfort. Jouki et al. demonstrated that the extraction circumstances had substantial effects on the mucilage yield and its protein content, viscosity, and antioxidant activity. They reported that at the optimum extraction conditions, 30% of antioxidant activity, 1470 mPA.s viscosity, and 3% of protein content were achieved with high emulsion stability that was conducted at 65 °C extraction temperature with the water/seed (W/S) ratio of 25:1 [36]. Abbastabar et al. investigated the rheological behavior of QSM and demonstrated that it shows non-Newtonian shear thinning flow. The dynamic rheological measurement exhibited that the addition of NaCl solution decreased the viscoelastic range, while the elastic modulus presented an increasing trend [37]. The results showed that QSG has high hydrodynamic volume and gelling ability in an aqueous solution. Moreover, their higher water absorption capability than arabinoxylans and other gums,makes them convenient for pH-sensitive on-off switching, which leads to the development of pH-responsive drug delivery systems [34]. Recently, Maroufi and Ghorbani reported the preparation of injectable chitosan/quince seed gum blends hydrogels to improve the properties of chitosan for investigating their curcumin release profiles. The results demonstrated that the increase in QSM ratio, thermal stability, swelling capacity, and degradation rate of hydrogels was improved [38]. Moreover, the scaffold application of quince seed hydrogels (QSH) crosslinked by glutaraldehyde was examined. It was shown that the QSH is a promising candidate for 3D cell culture and tissue engineering applications with its porous structure, high swelling ratio, and biocompatibility [39]. In another study, a new viscoelastic and fully biobased ink consisting of QSM and cellulose nanofibrils was prepared with excellent shear thinning and viscoelastic behavior and supporting cell growth by its nontoxic character [40].

The present study focuses on developing and characterizing an injectable composite hydrogel formulation from quinces seed gum and hyaluronic acid for dermal filler and intraarticular injections. The obtained formulations were examined physically and chemically and compared with conventional cross-linked HA hydrogels.

## 2. Materials and methods

### 2.1. Materials

A high molecular weight of hyaluronic acid powder (Mw = 800 kDa) was purchased from Shiseido Ltd. (Japan). 1,4-Butanediol diglycidyl ether (BDDE) and lyophilized bovine testicular hyaluronidase powder 400–1000 units/mg solid were purchased from Sigma-Aldrich Co. (USA). All other chemicals were prepared in-house (World Medicine Pharmaceutical Company, Türkiye.) Quince seeds from Türkiye were collected from the Marmara Region. For the amino acid determination, the L-amino acids were all supplied from Kyowa Hakko Bio Co.Ltd., and α-aminobutyric acid as an internal standard was purchased from Sigma (St. Louis, MO, USA). AccQ-Tag Eluent A was obtained from Waters Corp (Milford, MA, USA). ECF, and all other chemicals were of analytical grade from several suppliers.

### 2.2. Lyophilization of quince seed gum

Quince seeds (13.75 g) were treated with absolute ethanol to remove pigments and fats and then dried in an oven at 105 °C. The seeds soaked in 130 mL of deionized water at room temperature for 72 h. All the mucilage was then squeezed from the muslin bag to remove the marc. Anhydrous ethanol was added to the mucilage, precipitated, and filtered. The precipitated mucilage was spread on the watch glass and freeze-dried at −30 °C for 13 h in the Tofflon lyophilization device.

### 2.3. Fabrication of HA-QSG injectable hydrogels

**Figure 1:**
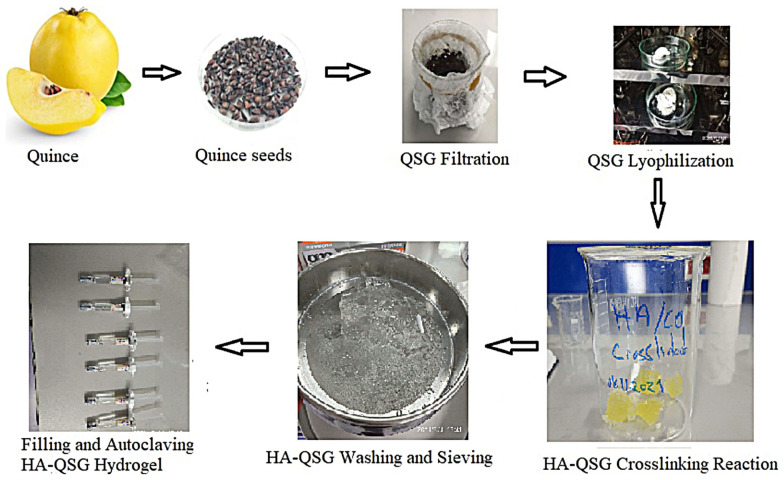
Production of the cross-linked HA-QSG injectable hydrogel pathway.

The injectable hydrogels were fabricated from biocompatible quince seed gum, hyaluronic acid, and BDDE with a weight ratio of 1:28:4. First, 5 mg of lyophilized quince seed gum was swollen in 1 mL of 0.25 M NaOH solution in a glass beaker. Next, 140 mg of hyaluronic acid powder was added and stirred carefully until a homogeneous mixture was obtained. Finally, 20 mg/mL of 1,4-Butanediol diglycidyl ether as a crosslinker was added to the mixture and stirred with a glass rod. HA-QSG-BDDE mixture was left to react in a thermostated water bath at 50 °C for 3.5 h. The cross-linked HA-QSG gel was neutralized with phosphate buffer solution by adding a 1 N HCl solution until a pH of approximately 7.0 was reached. The gel was kept in buffer solution for 24 h, and following this, the fully swollen gel was sieved through a 2-mm-mesh sieve. The sieved gel was washed six times in 350 mL of phosphate buffer to fully remove the unreacted BDDE. The obtained gel was divided into two parts. The first part was sieved through a 1000-μm-mesh stainless steel sieve to investigate the performance in the intraarticular knee synovial fluids application, and the other part of the gel was sieved through a 300-μm-mesh stainless steel sieve for the dermal filler application. The precipitated hydrogels in excess ethanol were dried under vacuum at room temperature for 24 h. Finally, the obtained gels with a 20 mg/mL concentration were filled into 1-mL syringes and sterilized in an air stream (Fedegari Autoclave) at 121 °C for 15 min. As a result of the autoclaving process, transparent hydrogels were obtained. The production of the composite hydrogel pathway is shown in Figure 1. For the comparison, the cross-linked HA hydrogels were prepared at the same weight ratio of HA:BDDE = 7:1.

### 2.4. Structural characterization

The FT-IR spectra of lyophilized QSG, HA hydrogel, and HA-QSG hydrogel were recorded on FT-IR spectroscopy (FTIR-SHIMADZU-Model: IRAffinity-1). The spectrum was obtained in a range of 400 to 4000 cm^−1^ at a resolution of 4 cm^−1^.

The HA and HA-QSG hydrogel samples were digested by bovine testicular hyaluronidase BTH enzyme solution (specific activity 400 IU/mg) at 37 °C overnight. The resulting solutions were centrifuged for 3 min at 3000 rpm using a Centurion Scientific centrifuge. The supernatant in each container was collected in a glass vial and lyophilized. The two samples were then dissolved in Deuterium oxide (D_2_O) for ^1^H NMR analysis. Analysis was carried out using 400 MHz ^1^H nuclear magnetic resonance spectroscopy (NMR) from Varian Mercury-VX 400 MHz BB.

### 2.5. Detection of residual unreacted BDDE

The residual BDDE after purification of hydrogels may cause many adverse effects such as carcinogenic effects and allergic reactions. Therefore, the amount of unreacted residual crosslinker in the hydrogels was evaluated by using gas chromatography (GC-SHIMADZU-Model: NEXIS GC-2030) and gas chromatography-mass spectroscopy (GC-MS-Agilent 5977B EI MS).

For the gas chromatography studies, the reference solution of 1,4-Butanediol diglycidyl ether in absolute ethanol (25 mg/10 mL) was prepared in a 50-mL flask. The flask was then completed to its volume with phosphate buffer solution (PBS). Subsequently, 1.0 mL of this solution was taken, and it was mixed with the PBS until it reached the final volume of 25 mL. It was filtered through a 0.45-μm PTFE filter and then vialed. Two parallel experiments were performed. Next, 1 g of gel sample was weighed into a 5-mL flask. Approximately 3 mL of PBS was added and vortexed for 2 min. It was then placed in an ultrasonic bath for 10 min. The volume was then adjusted to complete with PBS. The solution was filtered through a 0.45 μm PTFE filter and then vialed. The measurement was taken by injecting the phosphate buffer solution once, the standard solution 6 times, and the sample solution once into the column.

Chromatographic conditions: Column: DB-1 (30 m × 0.53 mm × 3.0 μm); Detector: FID, 250 °C; Carrier gas: nitrogen; Inlet temperature: 220 °C; Split ratio: 5:1; Flow rate: 3 mL/min; Flow mode: constant flow; Hydrogen flow rate: 30 mL/min; Dry air flow rate: 300 mL/min; Injection volume: 1 mL; Makeup flow rate: 25 mL/min (constant makeup flow rate); Column temperature: 180 °C.

For gas chromatography-mass spectroscopy studies, the hydrogel samples were vortexed in methanol and extracted. In addition, a standard solution of BDDE was prepared at a concentration of 10,000 mg/L (10,000 ppm) by diluting it in methanol. Both samples were loaded into the GC-MS instrument.

Chromatographic conditions: Column: Agilent; HP-5MS, 30 m × 0.25 mm × 0.25 μm; detector: MS, 280 °C; Signal speed: 20 Hz/0.01 min; Carrier gas: helium; Inlet temperature: 280 °C; Split ratio: 5:1; Flow rate: 2 mL/min; Flow mode: constant flow; Solvent delay time: 0 min; Mass scan range: full scan from m/z 20 to m/z 600; Column temperature: 70°C (1 min), ramped to 250 °C over 5 min; Injection volume: 2 mL.

### 2.6. The determination of amino acid content in the HA-QSG hydrogel using the HPLC method

For the degradation of hydrogel, a 1.5 g freeze-dried HA-QSG hydrogel sample was thoroughly mixed with hyaluronidase enzyme (400 IU/mg) solution at 37 °C in a thermostated water bath for 24 h.

The stock solutions of all amino acid standards (L-arginine, L-alanine L-valine, L-leucine, L-lysine, L-glycine, L-histidine, L-ornithine, L-phenylalanine, L-methionine, L-aspartic acid, L-glutamic acid, L-serine, L-threonine, L-proline) were prepared in water (50 mg/10 mL) [41]. Next, 150 μL of each amino acid stock solution was taken into a 50-mL flask containing 0.02 M HCl solution. Subsequently, 2.0 mL of internal standard was added, and the volume was adjusted to 50 mL with the same solution up to the mark on the flask. Finally, the solution was thoroughly mixed. As an internal solution, 25.8 mg α-aminobutyric acid was diluted by 0.1 M HCl solution in a 100 mL volumetric flask. After that, 1.5 g of sample solution was introduced into a 50-mL volumetric flask, followed by the addition of 2 mL of internal solution. The flask was then filled to its volume with 0.02 M HCl solution and thoroughly mixed.

The AccQ-Tag method was used as a precolumn derivatization. To reconstitute the derivative reagent, 1 mL of AccQ Fluor Reagent diluent (Vial 2B) was transferred into Vial 2A. The vial was then vortexed for 10 s. After that, AccQ-Fluor Reagent Powder (Vial 2A) was placed in an oven and allowed to dissolve for 10 min.

10 μL of the sample and the standard solution were each transferred into a volumetric flask, followed by the addition of 70 μL of AccQ Fluor Borate Buffer. The mixture was thoroughly mixed, and then 20 μL of derivatization reagent (Vial 2A) was added. The flask was then placed in an oven preheated to 55 °C and left for 10 min. The vial was removed from the oven and vortexed for 10 sec. Subsequently, it was allowed to cool to room temperature, approximately 10 minutes, before being injected into the system.

Analysis was performed using a Waters E2695 model liquid chromatography with a fluorescence detector. Mobile Phase A: 200 mL of Acc.Tag Eluent A solution diluted to 2 L with distilled water; Mobile Phase B: acetonitrile; Mobile Phase C: pure water column conditioning: The column temperature was set to 37 °C. The column was conditioned for 5 min with 60% mobile phase B/40% mobile phase C at 1.0 mL/min flow. After that, it was conditioned for 9 min with 100% mobile phase A at a flow of 1.0 mL/min.

### 2.7. Equilibrium swelling ratio

The lyophilized gel sample was immersed in deionized water and was taken out when it reached the equilibrium state. The excess water on the surface was removed using a filter. Measurements were performed three times, and the averages were calculated. The equilibrium swelling ratio (SR) of the hydrogels was calculated using Eq. (1).


Eq. (1) $$SR\;\left(\frac{g}{g}\right)=\frac{\left(Ww\right)}{\left(Wd\right)}$$

where Ww and Wd indicate the hydrogels’ wet and dry weights, respectively.

### 2.8. Particle size distribution

The hydrogels were crushed through 1000-μm and 300-μm stainless steel sieves and the particle size measurement was performed in 0.9% NaCl solution in a particle size measuring device (Malvern Master Sizer 2000).

### 2.9. Rheology measurements

A Physica MCR302 oscillatory rheometer (Anton Paar, Germany) that has a parallel plate geometry, a 25 mm plate diameter, a 1.0 mm gap, and a Peltier temperature control was used for the experiments. The test temperature was 25 °C. The oscillation frequency sweep tests were conducted over a frequency range of 0.1 to 10 Hz with a strain value of 0.1%, which falls within the linear viscoelastic limits according to the amplitude sweep test (Figure S1).

### 2.10. Scanning electron microscopy (SEM)

The fractured surface structures of HA-QSG and HA hydrogels were imaged using a scanning electron microscope (Philips-FEI XL30 ESEM-FEG). SEM images showed information about the porosity and scaffold connectivity of hydrogels. All samples were lyophilized before measurement and coated under vacuum with platinum using an ion sputter prior. The morphological features of HA/QSG and HA hydrogels were compared by analyzing SEM images at identical magnification (10×, 50×, SEI, and 15 kv).

## 3. Results and discussion

### 3.1. Structural characterization

**Figure 2:**
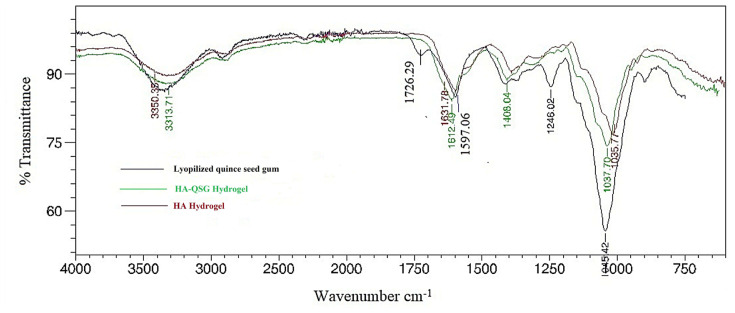
FT-IR spectra of lyophilized quince seed gum, HA-QSG hydrogel, and HA hydrogel.

The HA-QSG hydrogel was characterized using FTIR analysis and compared with HA gel and lyophilized quince seeds gum in Figure 2. As shown in Figure 2, the characteristic peaks belonging to QSG at 1597 cm^−1^ (asymmetric str. groups of carboxylate anion) and 1727 cm^−1^ (stretching groups of –COOH) were observed [42]. The characteristic O-H and N-H str. vibration peaks related to HA were observed at 3314 cm^−1^. The peak at around 2950 cm^−1^ is attributed to aliphatic C-H str. vibrations. The peaks at 1612 cm^−1^ and 1408 cm^−1^ refer to the asym and symm. str. vibrations of C = O groups of the planar carboxyl groups in the hyaluronate. Additionally, the peaks around 1155–1044 cm^−1^ are attributed to C-O-C and C-OH groups. In the HA-QSG gels, mainly the same absorption peaks were observed. The peaks of the QSG could not be distinguished because they overlapped with the HA peaks.

The hydrogel structure was confirmed using the ^1^H and ^13^C NMR spectra. As can be seen in Figures S2a and S2b (please see the Supplementary Information section at the end), the resonance peaks for HA hydrogel were observed at 1.90 ppm (CH_3_ group in N-acetyl group of HA), 4.35–4.50 ppm (O-CH-O of HA), 3.20–3.90 ppm (sugar ring H of HA), and 1.5 ppm (CH_2_-CH_2_-O of BDDE). The NMR results indicate that the synthesis of the BDDE crosslinking reaction of HA hydrogel was successful, and the absence of the characteristic peaks of unreacted BDDE at 2.70–2.85 ppm suggests that residual BDDE was effectively removed. Solvent used was D2O (4.65 ppm) [43]. The ^1^H-NMR spectrum of the cross-linked HA-QSG revealed new peaks at 3.0 ppm and 5.10 ppm which may correspond to a 4-O-Me-glucuronic acid moiety of QSG [44]. Moreover, the ^13^C NMR spectrum of HA-QSG (Figure S3) indicated its highly branched structure. The resonance signals for carbonyl groups of the D-glucuronic acid were observed at around 176 ppm. The signals at 100.5 and 103 ppm were assigned to glycosyl residues. The carbon signal for the methoxyl group was at 60.80 ppm. The NMR data were in agreement with those obtained for polysaccharides [31].

### 3.2. Residual BDDE analysis

**Figure 3:**
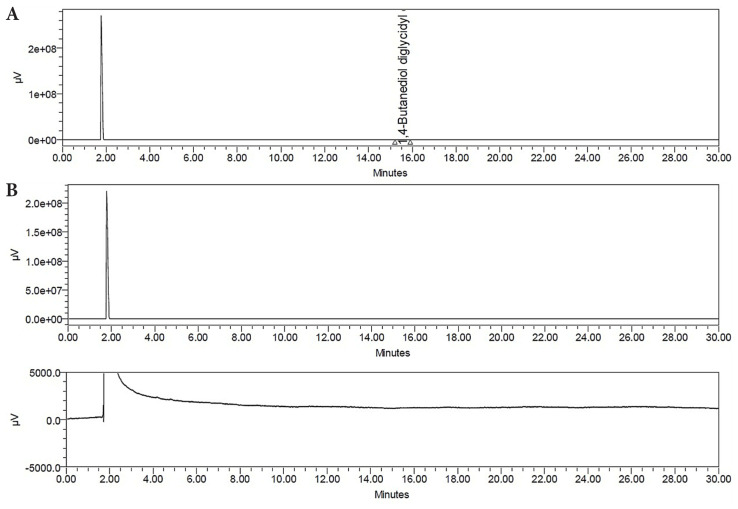
The residual BDDE analysis by GC. a) Standard reference peak of BDDE, b) HA-QSG hydrogel.

**Figure 4:**
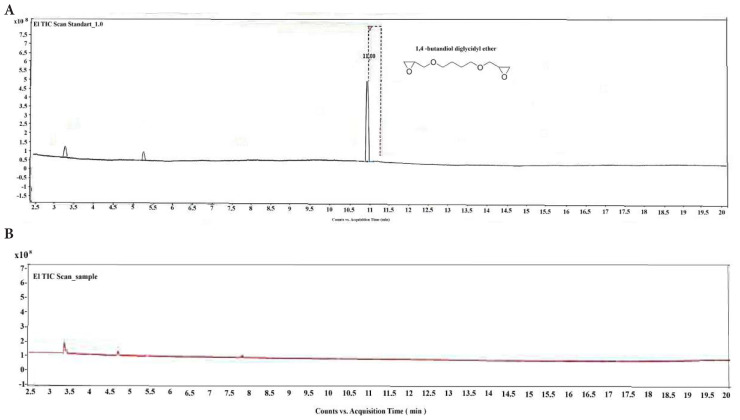
The residual BDDE analysis by GC-MS. a) Standard reference peak of BDDE, b) HA-QSG hydrogel.

The presence of residual BDDE was assessed by using GC and GC-MS analyses, as shown in Figures 3 and 4. According to the Food and Drug Administration (FDA), the residual BDDE is considered nontoxic when it is lower than 2 ppm [44]. In Figure 3, a peak of reference BDDE solution (0.2 wt/v %) was observed at a retention time (RT) of 15.62 min. The HA-QSG hydrogel sample was analyzed and there was no peak observed in the chromatogram indicating successful separation of BDDE.

In Figure 4, the residual BDDE analysis results using the GC-MS technique are shown. A reference standard BDDE peak was found with a retention time of 11.25 min. After washing and autoclaving HA-QSG hydrogel, any detection peak for the unreacted BDDE was not found via GC-MS analysis. It is concluded that the amount of unreacted BDDE in cross-linked HA-QSG hydrogels is maintained at an undetected amount, which results in a residual level of unreacted BDDE of <2 ppm, which satisfies FDA requirements.

### 3.3. Free amino acid analysis

As the building blocks of proteins, amino acids are crucial for the physiological turnover of tissues. Amino acids are known to enhance the production of proteins, according to several in vivo and in vitro studies. For the synthesis of collagen polypeptide chains, glycine, L-proline, and L-alanine are primarily used, while L-serine, L-lysine, L-leucine, and L-isoleucine are used to a lesser extent. However, it has been reported that glycine, L-valine, L-alanine, and L-proline are necessary for the synthesis of elastin [45].

**Figure 5:**
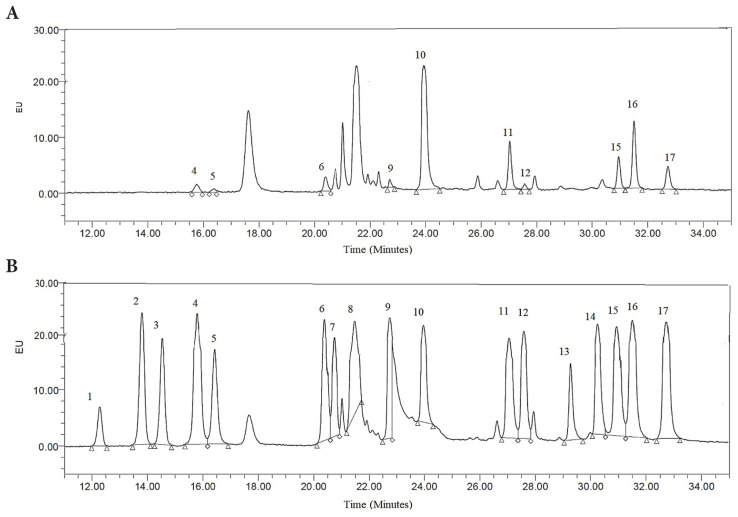
Chromatogram of free amino acids a) in standard solution, b) in HA-QSG hydrogel sample. (1) L-aspartic acid, (2) L-serine, (3) L-glutamic acid, (4) glycine, (5) L-histidine, (6) L-arginine, (7) L-threonine, (8) L-alanine, (9) L-proline, (10) α-aminobutyric acid, (11) L-valine, (12) L-methionine, (13) L-ornithine, (14) L-lysine, (15) L-isoleucine, (16) L-leucine, (17) L-phenylalanine.

The chromatographic separation of amino acid standard peaks and the amino acids in the HA-QSG hydrogel sample are shown in Figures 5a and 5b, respectively. Since the chromatographic separation of the amino acid peaks of both the standard sample and the HA-QSG hydrogel was quite good, amino acids were successfully identified. In the standard sample, all 17 amino acids, including the internal standard (a-aminobutyric acid), were easily recognized.

Leucine, arginine, and valine had the highest concentrations of free amino acids in the HA-QSG hydrogel, which was then followed by phenylalanine, isoleucine, glycine, proline, histidine, and methionine (Table 1). It is well known that hyaluronic acid itself helps the skin’s extracellular matrix molecule and water content. On the other hand, amino acids in QSG can enhance hyaluronic acid’s capabilities and work well with intradermal microinjections to rejuvenate the skin.

### 3.4. Swelling ratio

The swelling of hydrogel is crucial due to its use in wound healing, drug delivery, and of course, TE applications because it characterizes the ability of hydrogels to absorb body fluids and control the transfer of cell nutrients and metabolites. The swelling capacity of hydrogels depends on pore size, cross-linking density, and polymer-polymer and polymer-solvent interactions. Hydrogels reached equilibrium swelling in 24 h. In comparison to HA, HA-QSG hydrogels showed a slightly lower equilibrium swelling ratio. The swelling ratio of the HA-QSG hydrogel in distilled water was 30.0 ± 1.18, whereas the average for HA gels was 32.6 ± 2.33. Due to the branched nature of QSG, composite gels containing QSG form more entangled structures than those containing HA, and the high cross-link density and smaller pore diameter are the cause of the low swelling.

### 3.5. Particle size distribution

After washing and neutralizing the HA-QSG hydrogels, they were manually sieved twice though a 1000-μm sieve for the intraarticular injection and a 300-μm sieve for the dermal filler injection. Results for particle size measurements are summarized in Table 2.

**Figure 6:**
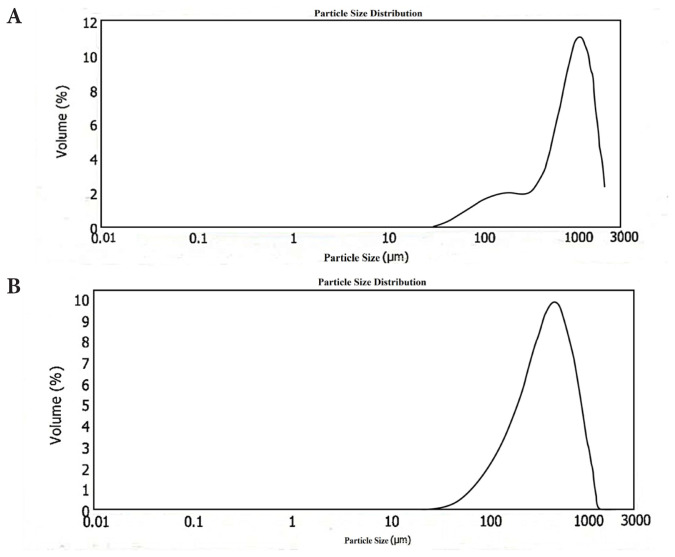
Particle size distribution of a) the HA-QSG hydrogel for dermal filler injection. b) the HA-QSG hydrogel for intraarticular injection.

In particular, Figures 6a and 6b show the size distribution profiles for the HA-QSG hydrogels. The gel prepared for dermal filler injection application was found to have a diameter range of 83–600 μm and the gel prepared for intraarticular injection application to have a diameter range of 148–1499 μm. The majority of particles Dv (90% volume fraction) were smaller than 611 μm and 1499 μm, respectively. Values of mean volume diameter Dv [4.3] and mean surface diameter Dv [3.2] were also reported to be about 318 μm and 833 μm, respectively (Table 2).

### 3.6. Rheology

Injectable scaffolds based on HA hydrogels are significant biomaterials for tissue engineering. Given that hydrogels are considered viscoelastic materials, they might display viscous or elastic behavior depending on the conditions. Rheological properties of injectable hydrogels include elasticity, viscosity, and plasticity. The rheological characteristics of injectable hydrogels are therapeutically important because they determine how the hydrogel behaves after injection. Complex viscosity (η*) and elastic modulus (G′) are considered the most important rheological characteristics for injectable hydrogels. The capacity of a gel to withstand shearing forces within a tissue after injection is measured by complex viscosity, whereas elastic modulus assesses the stiffness of a hydrogel and its interactions. These factors define how effectively it resists tension forces caused by injected region movement following injection [46].

**Figure 7:**
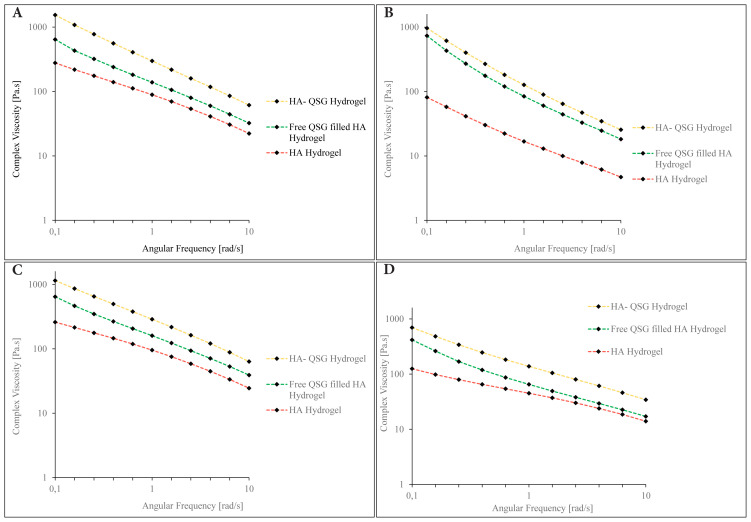
Complex viscosity dependence of the HA-QSG hydrogel, free QSG-filled HA hydrogel, and HA hydrogels on angular frequency a) for intraarticular injection before steam sterilization, b) for intraarticular injection after steam sterilization, c) for dermal filler injection before steam sterilization, d) for dermal filler injection after steam sterilization.

Hydrogels made from lyophilized QSG can be used in tissue engineering procedures requiring higher stiffness, such as bone regeneration. The viscoelastic properties of HA-QSG hydrogels and HA hydrogels for intraarticular and dermal injection applications as a function of the oscillation frequency are shown in Tables 3 and 4, respectively. (The detailed data are provided in Tables S1–S4.) For comparison, free QSG-filled HA hydrogels were also studied, and the results were included. The relative increase in elastic modulus (G′) over loss modulus (G″) caused the gels to become stronger and harder. The elastic modulus of the HA-QSG hydrogel is significantly greater than both the free QSG-filled HA gel and the HA hydrogels. The significantly increased elastic modulus (G′) indicates that the covalent crosslinking between QSG and HA chains is effective, and the gel has a high crosslinking density. Harder gels are generally preferable in bone and joint areas, as is well known. Besides their physical strength, they also show larger particle sizes with low swelling and resistance to enzymatic degradation. The analysis of the results revealed that the gel particles with a larger diameter showed higher mechanical resistance, as well as complex viscosity. On the contrary, soft gels have poor physical strength, low adhesion resistance, small particle sizes, and high swelling properties in the water, and are easily degraded by enzymatic degradation. They are mostly used to remove wrinkles on the face [47,48]. Smaller-sized gels prepared for dermal filler injection showed lower elastic modulus and complex viscosity compared to gels prepared for intraarticular injection. In addition, it was found that thermal sterilizing somewhat reduced the elastic modulus and the complex viscosity. However, the decrease in these values does not constitute a disadvantage in terms of its use as an injection material. Figure 7 illustrates the dependence of the complex viscosity versus the angular frequency of the QSG-HA hydrogels. The decrease in complex viscosity with angular frequency has been is attributed to the weakening and breaking of attractive forces. This behavior illustrates the shear-thinning pseudoplastic behavior of the hydrogels [49,50]. The complex viscosity of intraarticular injectable gels was quite a bit higher than that of dermal filler injectable ones. The higher viscosity can be explained by intermolecular interactions, such as crosslinking and hydrogen bonding, between the polymer chains. Similar behavior was observed for free QSG-filled HA hydrogel and HA hydrogels.

### 3.7. Microstructural analysis with SEM

**Figure 8:**
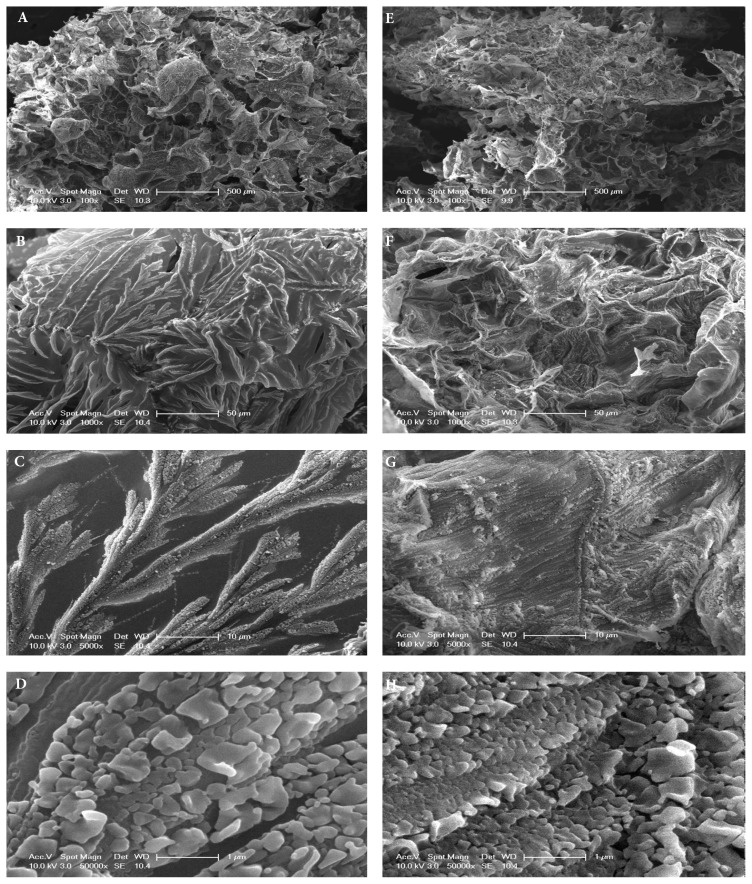
Sem micrographs of HA (a–b–c–d) and HA-QSG (e–f–g–h) hydrogels at 100x 1000x 5000x 50.000x magnifications.

By using SEM analysis, the microarchitecture of hydrogels, such as porosity, pore diameters, and interconnectivity of pores, were assessed. All hydrogels were lyophilized before the SEM analysis. As can be seen in Figures 8a and 8b at 100× magnification, HA-QSG and HA hydrogels demonstrated a very porous microstructure, characterized by a fibrous network configuration [51]. For HA hydrogels, the pore size was under 250 mm and they were more regular, and each pore was frequent and interconnected. Despite this, the pore size distribution of the HA-QSG hydrogel remained below 200 μm, with a frequent observation of a closed pore structure. Additionally, the pore sizes match the equilibrium swelling ratios of the gels. The ESR of the QSG-HA hydrogel with denser and closed pores was lower. When the polymer walls were magnified at 1000× and 5000×, distinct structures were observed in the surface morphology of both hydrogels (Figures 8c–8f). Particularly in the HA gel, it was observed that the particles were spread in the form of a more uniform crystal formation. At a higher magnification of 50,000× (Figures 8g and 8h), the gel wall surface investigation showed that the highly ordered sequence of polymer particles less than 1 mm was observed. It can be said that the crosslinking of QSG with HA results in a more homogenous morphology and inhibits HA from aligning into crystals in the wall regions of the gel. This finding supports the increase in the elastic modulus of the QSG-HA hydrogel.

## 4. Conclusion

We presented a new injectable hyaluronic acid composition approach using a natural polymer quince seed gum. When the amino acid content of QSG is examined, it was detected that about nine free amino acids appear with good separation, as well as consistency in the retention times. Leucine, arginine, and valine had the highest concentrations of free amino acids in the HA-QSG hydrogel. Quince seed gum plays an important role with regard to the elastic module of the cross-linked hydrogel. The results clearly suggest that the HA-QSG hydrogels exhibited high elastic modulus and complex viscosity, which are especially superior candidates for injectable applications due to their highly tunable properties. This study serves as a proof-of-concept exploration aimed at developing a novel biomaterial for dermal and intra-articular filler applications, with the potential for therapeutic use and eventual clinical investigation.

## Supplementary Data

Figure S1 LVE limit graph of HA-QSH hydrogel.

Figure S2a ^1^H-NMR spectra of HA-QSG hydrogel.

Figure S2b ^1^H-NMR spectra of HA hydrogel

Figure S3 ^13^C -NMR spectra of HA-QSG hydrogel structure.

**Table S1:** Intraarticular hydrogels before steam sterilization rheology data.

Sample no. 1	Meas. pts.	Angular frequency [Rad/s]	Storage modulus [Pa]	Frequency [Hz]	Loss modulus [Pa]	Damping factor [ 1]	Complex viscosity [Pa.s]	Deflection angle [mrad]	Torque [μNm]	Temperature [°C]
**HA-QSG hydrogel**	1	100	183	15.9	272	1.48	3.28	0.801	10	25
	2	63.1	588	10	296	0.504	10.4	0.801	20.2	25
	3	39.8	695	6.34	298	0.428	19	0.801	23.2	25
	4	25.1	683	4	292	0.427	29.6	0.801	22.8	25
	5	15.8	626	2.52	279	0.446	43.3	0.801	21	25
	6	10	555	1.59	262	0.473	61.4	0.799	18.8	25
	7	6.31	482	1	242	0.501	85.5	0.8	16.5	25
	8	3.98	414	0.634	218	0.526	118	0.8	14.3	25
	9	2.51	354	0.4	192	0.543	160	0.8	12.3	25
	10	1.58	303	0.252	167	0.549	218	0.8	10.6	25
	11	1	261	0.159	143	0.549	298	0.8	9.11	25
	12	0.631	226	0.1	122	0.538	407	0.799	7.85	25
	13	0.398	197	0.0634	104	0.526	559	0.799	6.81	25
	14	0.251	175	0.04	87.1	0.499	777	0.798	5.96	25
	15	0.158	154	0.0252	75.2	0.488	1080	0.798	5.24	25
	16	0.1	140	0.0159	63.7	0.455	1540	0.794	4.68	25

**Table:** 

Sample no. 2	Meas. pts.	Angular frequency [Rad/s]	Storage modulus [Pa]	Frequency [Hz]	Loss modulus [Pa]	Damping factor [ 1]	Complex viscosity [Pa.s]	Deflection angle [mrad]	Torque [μNm]	Temperature [°C]
**Free QSG filled HA hydrogel**	1	100	0	15.9	288	1	2.88	0.8	8.82	25
	2	63.1	243	10	250	1.03	5.52	0.801	10.7	25
	3	39.8	289	6.34	208	0.718	8.95	0.801	10.9	25
	4	25.1	326	4	203	0.623	15.3	0.8	11.8	25
	5	15.8	308	2.52	190	0.618	22.8	0.8	11.1	25
	6	10	271	1.59	175	0.645	32.3	0.799	9.87	25
	7	6.31	231	1	158	0.684	44.3	0.798	8.54	25
	8	3.98	192	0.634	141	0.735	59.9	0.798	7.27	25
	9	2.51	158	0.4	124	0.784	79.8	0.798	6.12	25
	10	1.58	129	0.252	107	0.831	106	0.797	5.11	25
	11	1	105	0.159	91.3	0.869	139	0.798	4.25	25
	12	0.631	85.6	0.1	76.8	0.898	182	0.796	3.5	25
	13	0.398	70.8	0.0634	64.3	0.908	240	0.796	2.91	25
	14	0.251	60.3	0.04	53.6	0.889	321	0.794	2.45	25
	15	0.158	51.9	0.0252	44.3	0.854	431	0.795	2.08	25
	16	0.1	51.8	0.0159	38.2	0.739	644	0.788	1.94	25
	f=4.451 Hz, G′=G″ = 156.2 Pa (Cross over), ω(omega)=27.97 rad/s

**Table:** 

Sample no. 3	Meas. pts.	Angular frequency [Rad/s]	Storage modulus [Pa]	Frequency [Hz]	Loss modulus [Pa]	Damping factor [ 1]	Complex viscosity [Pa.s]	Deflection angle [mrad]	Torque [μNm]	Temperature [°C]
**HA hydrogel**	1	100	0	15.9	432	1	4.32	0.801	13.2	25
	2	63.1	33	10	148	4.48	2.4	0.801	4.64	25
	3	39.8	186	6.34	145	0.78	5.92	0.801	7.22	25
	4	25.1	222	4	139	0.627	10.4	0.801	8.03	25
	5	15.8	213	2.52	129	0.603	15.7	0.8	7.63	25
	6	10	189	1.59	118	0.627	22.3	0.799	6.81	25
	7	6.31	160	1	107	0.669	30.6	0.798	5.89	25
	8	3.98	132	0.634	95.8	0.724	41	0.799	4.99	25
	9	2.51	107	0.4	83.9	0.787	54	0.799	4.15	25
	10	1.58	84.3	0.252	71.9	0.853	69.9	0.799	3.39	25
	11	1	65.6	0.159	60.3	0.92	89.1	0.799	2.73	25
	12	0.631	50.7	0.1	49.2	0.97	112	0.8	2.16	25
	13	0.398	39.4	0.0634	39.4	1	140	0.8	1.71	25
	14	0.251	30.9	0.04	31.2	1.01	175	0.8	1.34	25
	15	0.158	24.5	0.0252	24.6	1	219	0.8	1.06	25
	16	0.1	19.8	0.0159	19.5	0.986	278	0.8	0.851	25
	f=6.766/0.06418/0.02383 Hz, G′=G″=145.3/39.67/23.90 Pa, ω(omega)=42.51/0.4033/0.1497 rad/s, η (eta)=380.87									

**Table S2:** Intraarticular hydrogels after steam sterilization rheology data.

Sample no. 1	Meas. pts.	Angular frequency [Rad/s]	Storage modulus [Pa]	Frequency [Hz]	Loss modulus [Pa]	Damping factor [ 1]	Complex viscosity [Pa.s]	Deflection angle [mrad]	Torque [μNm]	Temperature [°C]
**HA-QSG hydrogel**	1	100	0	15.9	326	1	3.26	0.8	10	25
	2	63.1	171	10	229	1.34	4.53	0.8	8.75	25
	3	39.8	255	6.34	215	0.845	8.38	0.8	10.2	25
	4	25.1	268	4	191	0.713	13.1	0.8	10.1	25
	5	15.8	246	2.52	163	0.665	18.6	0.8	9.04	25
	6	10	215	1.59	137	0.638	25.5	0.799	7.81	25
	7	6.31	187	1	113	0.605	34.6	0.8	6.68	25
	8	3.98	163	0.634	92.6	0.569	47	0.799	5.73	25
	9	2.51	144	0.4	75.1	0.523	64.5	0.799	4.96	25
	10	1.58	128	0.252	61.1	0.476	89.8	0.799	4.35	25
	11	1	116	0.159	49.5	0.425	127	0.798	3.87	25
	12	0.631	108	0.1	39.9	0.371	182	0.797	3.5	25
	13	0.398	102	0.0634	32.5	0.320	268	0.796	3.25	25
	14	0.251	97	0.04	27.8	0.286	401	0.795	3.07	25
	15	0.158	94.2	0.0252	24.8	0.264	615	0.797	2.97	25
	16	0.1	92.4	0.0159	28.2	0.305	966	0.792	2.93	25
	f=7.489 Hz, G′=G″ =220.3 Pa (Cross over), ω(omega)=47.05 rad/s, η (eta)=24.731 Pa.s

**Table:** 

Sample no. 2	Meas. pts.	Angular frequency [Rad/s]	Storage modulus [Pa]	Frequency [Hz]	Loss modulus [Pa]	Damping factor [ 1]	Complex viscosity [Pa.s]	Deflection angle [mrad]	Torque [μNm]	Temperature [°C]
**Free QSG filled HA hydrogel**	1	100	0	15.9	413	1	4.13	0.8	12.6	25
	2	63.1	53.5	10	214	3.99	3.49	0.8	6.74	25
	3	39.8	125	6.34	169	1.35	5.28	0.801	6.45	25
	4	25.1	167	4	152	0.913	9	0.8	6.92	25
	5	15.8	162	2.52	131	0.807	13.2	0.8	6.39	25
	6	10	144	1.59	111	0.77	18.2	0.799	5.57	25
	7	6.31	125	1	93.2	0.747	24.7	0.798	4.75	25
	8	3.98	106	0.634	77.1	0.727	32.9	0.798	4	25
	9	2.51	91	0.4	63.6	0.700	44.2	0.797	3.39	25
	10	1.58	80	0.252	52.6	0.657	60.4	0.799	2.93	25
	11	1	72.1	0.159	43.7	0.607	84.3	0.798	2.58	25
	12	0.631	66.5	0.1	36.6	0.551	120	0.797	2.31	25
	13	0.398	62.7	0.0634	31.6	0.503	176	0.795	2.14	25
	14	0.251	61.7	0.04	28.5	0.461	271	0.792	2.06	25
	15	0.158	62.5	0.0252	27.7	0.443	431	0.789	2.07	25
	16	0.1	66.9	0.0159	30.3	0.453	735	0.789	2.22	25
	f=9.648 Hz, G′=G″=246.3 Pa (Cross over), ω(omega)=60.62 rad/s, η (eta)=1.956.6 Pa.s

**Table:** 

Sample no. 3	Meas. pts.	Angular frequency [Rad/s]	Storage modulus [Pa]	Frequency [Hz]	Loss modulus [Pa]	Damping factor [ 1]	Complex viscosity [Pa.s]	Deflection angle [mrad]	Torque [μNm]	Temperature [°C]
**HA hydrogel**	1	100	0	15.9	720	1	7.2	0.801	22	25
	2	63.1	0	10	245	1	3.88	0.801	7.5	25
	3	39.8	0	6.34	82.9	1	2.08	0.801	2.54	25
	4	25.1	8.86	4	55.6	6.28	2.24	0.801	1.73	25
	5	15.8	26.9	2.52	45.6	1.7	3.34	0.801	1.62	25
	6	10	29.3	1.59	36.6	1.25	4.69	0.8	1.44	25
	7	6.31	26	1	28.7	1.1	6.15	0.8	1.19	25
	8	3.98	22	0.634	22.3	1.01	7.87	0.799	0.957	25
	9	2.51	18.5	0.4	17.2	0.928	10	0.799	0.771	25
	10	1.58	15.7	0.252	13.3	0.845	13	0.799	0.628	25
	11	1	13.4	0.159	10.2	0.763	16.8	0.8	0.515	25
	12	0.631	11.6	0.1	7.96	0.687	22.3	0.799	0.43	25
	13	0.398	10.2	0.0634	6.33	0.621	30.2	0.798	0.367	25
	14	0.251	9.07	0.04	5.11	0.564	41.4	0.798	0.318	25
	15	0.158	8.13	0.0252	4.2	0.516	57.8	0.798	0.279	25
	16	0.1	7.33	0.0159	3.49	0.476	81.2	0.797	0.248	25
	f=0.5870 Hz, G′=G″=21.35 Pa (cross over), ω(omega)=3.688 rad/s									

**Table S3:** Dermal filler hydrogels before steam sterilization rheology data.

Sample no. 1	Meas. pts.	Angular frequency [Rad/s]	Storage modulus [Pa]	Frequency [Hz]	Loss modulus [Pa]	Damping factor [ 1]	Complex viscosity [Pa.s]	Deflection angle [mrad]	Torque [μNm]	Temperature [°C]
**HA-QSG hydrogel**	1	100	200	15.9	264	1.32	3.32	0.801	10.2	25
	2	63.1	607	10	292	0.481	10.7	0.801	20.6	25
	3	39.8	715	6.34	297	0.416	19.4	0.801	23.7	25
	4	25.1	704	4	296	0.42	30.4	0.801	23.4	25
	5	15.8	645	2.52	288	0.446	44.6	0.801	21.6	25
	6	10	571	1.59	276	0.483	63.4	0.8	19.4	25
	7	6.31	493	1	261	0.529	88.3	0.799	17	25
	8	3.98	417	0.634	240	0.575	121	0.8	14.7	25
	9	2.51	348	0.4	217	0.623	163	0.8	12.5	25
	10	1.58	288	0.252	191	0.666	218	0.8	10.6	25
	11	1	236	0.159	166	0.702	288	0.8	8.82	25
	12	0.631	193	0.1	141	0.728	379	0.799	7.31	25
	13	0.398	159	0.0634	118	0.741	497	0.8	6.05	25
	14	0.251	132	0.04	97.6	0.741	652	0.799	5.01	25
	15	0.158	110	0.0252	80.8	0.732	863	0.799	4.18	25
	16	0.1	93.6	0.0159	67	0.715	1150	0.799	3.51	25
	f=14.03 Hz, G′=G″=271.6 Pa (cross over), ω (omega)= 88.14 rad/s (Cross over rad/s), η (eta)=1.499.6 Pa.s

**Table:** 

Sample no. 2	Meas. pts.	Angular frequency [Rad/s]	Storage modulus [Pa]	Frequency [Hz]	Loss modulus [Pa]	Damping factor [ 1]	Complex viscosity [Pa.s]	Deflection angle [mrad]	Torque [μNm]	Temperature [°C]
**Free QSG filled HA hydrogel**	1	100	0	15.9	316	1	3.16	0.8	9.67	25
	2	63.1	376	10	325	0.87	7.87	0.8	15.2	25
	3	39.8	369	6.34	263	0.711	11.4	0.801	13.9	25
	4	25.1	401	4	258	0.643	19	0.8	14.6	25
	5	15.8	371	2.52	242	0.652	27.9	0.8	13.5	25
	6	10	321	1.59	222	0.69	39.1	0.799	11.9	25
	7	6.31	270	1	200	0.742	53.2	0.798	10.2	25
	8	3.98	221	0.634	177	0.803	71.1	0.798	8.64	25
	9	2.51	178	0.4	154	0.866	93.7	0.798	7.19	25
	10	1.58	142	0.252	132	0.931	123	0.798	5.93	25
	11	1	113	0.159	112	0.99	160	0.798	4.87	25
	12	0.631	90.1	0.1	93.6	1.04	206	0.798	3.97	25
	13	0.398	72.2	0.0634	77.4	1.07	266	0.798	3.23	25
	14	0.251	59.5	0.04	63.8	1.97	348	0.897	2.66	25
	15	0.158	51.2	0.0252	53.1	1.04	465	0.797	2.24	25
	16	0.1	46.4	0.0159	44.8	0.966	645	0.793	1.95	25
	f: 2.800 Hz, G′=G″ =144.2 Pa (Cross over), ω (omega)= 17.59 rad/s (cross over rad/s), η (eta)= 4.006.3 (carreau-y)

**Table:** 

Sample no. 3	Meas. pts.	Angular frequency [Rad/s]	Storage modulus [Pa]	Frequency [Hz]	Loss modulus [Pa]	Damping factor [ 1]	Complex viscosity [Pa.s]	Deflection angle [mrad]	Torque [μNm]	Temperature [°C]
**HA hydrogel**	1	100	0	15.9	432	1	4.32	0.8	13.2	25
	2	63.1	56.8	10	167	2.94	2.79	0.801	5.4	25
	3	39.8	212	6.34	162	0.764	6.71	0.801	8.18	25
	4	25.1	246	4	156	0.632	11.6	0.8	8.93	25
	5	15.8	235	2.52	144	0.613	17.4	0.8	8.45	25
	6	10	206	1.59	132	0.542	24.5	0.8	7.5	25
	7	6.31	174	1	120	0.687	33.5	0.798	6.45	25
	8	3.98	143	0.634	107	0.748	44.8	0.798	5.44	25
	9	2.51	114	0.4	93.3	0.819	58.6	0.799	4.5	25
	10	1.58	89.1	0.252	80.1	0.899	75.6	0.799	3.66	25
	11	1	68.2	0.159	67.1	0.984	95.6	0.8	2.93	25
	12	0.631	51.4	0.1	54.8	1.07	119	0.8	2.3	25
	13	0.398	38.4	0.0634	43.7	1.14	146	0.802	1.78	25
	14	0.251	28.8	0.04	34	1.18	177	0.802	1.37	25
	15	0.158	21.9	0.0252	26.1	1.19	215	0.803	1.04	25
	16	0.1	17	0.0159	19.7	1.16	260	0.802	0.799	25
	f=6.947/0.1453 Hz.G′=G″=163.1/63.47 Pa (Cross over), ω (omega)=43.65/0.9132 rad/s, η (eta)=330.6 Pa.s (carreau-y)									

**Table S4:** Dermal filler hydrogels after steam sterilization rheology data.

Sample no. 1	Meas. pts.	Angular frequency [Rad/s]	Storage modulus [Pa]	Frequency [Hz]	Loss modulus [Pa]	Damping factor [ 1]	Complex viscosity [Pa.s]	Deflection angle [mrad]	Torque [μNm]	Temperature [°C]
**HA-QSG hydrogel**	1	100	0	15.9	277	1	2.77	0.8	8.48	25
	2	63.1	254	10	250	0.982	5.65	0.8	10.9	25
	3	39.8	365	6.34	241	0.661	11	0.8	13.4	25
	4	25.1	372	4	228	0.614	17.4	0.8	13.4	25
	5	15.8	336	2.52	208	0.619	25	0.801	12.1	25
	6	10	288	1.59	186	0.647	34.3	0.8	10.5	25
	7	6.31	241	1	164	0.68	46.1	0.796	8.87	25
	8	3.98	198	0.634	141	0.712	61.1	0.8	7.44	25
	9	2.51	163	0.4	119	0.729	80.2	0.8	6.17	25
	10	1.58	134	0.252	98.3	0.732	105	0.8	5.09	25
	11	1	112	0.159	79.8	0.712	138	0.8	4.21	25
	12	0.631	95.4	0.1	64.5	0.676	183	0.799	3.52	25
	13	0.398	83	0.0634	52	0.626	246	0.798	2.99	25
	14	0.251	74.5	0.04	42	0.564	340	0.797	2.61	25
	15	0.158	68.2	0.0252	34.6	0.507	482	0.796	2.33	25
	16	0.1	63	0.0159	29.6	0.469	696	0.794	2.12	25
	f=14.03 Hz, G′=G″=271.6 Pa (cross over), ω(omega)=:88.14 rad/s, η (eta)= 1.499.6 Pa.s

**Table:** 

Sample no. 2	Meas. pts.	Angular frequency [Rad/s]	Storage modulus [Pa]	Frequency [Hz]	Loss modulus [Pa]	Damping factor [ 1]	Complex viscosity [Pa.s]	Deflection angle [mrad]	Torque [μNm]	Temperature [°C]
**Free QSG filled HA hydrogel**	1	100	0	15.9	394	1	3.94	0.8	12.1	25
	2	63.1	120	10	277	2.31	4.78	0.8	9.24	25
	3	39.8	109	6.34	177	1.63	5.23	0.8	6.38	25
	4	25.1	148	4	161	1.09	8.73	0.8	6.72	25
	5	15.8	143	2.52	140	0.976	12.6	0.799	6.11	25
	6	10	124	1.59	118	0.953	17.1	0.799	5.24	25
	7	6.31	103	1	0.1	0.963	22.6	0.798	4.37	25
	8	3.98	83.9	0.634	81.6	0.973	29.4	0.798	3.58	25
	9	2.51	68.6	0.4	66.5	0.97	38	0.797	2.92	25
	10	1.58	56.5	0.252	54	0.956	49.3	0.798	2.39	25
	11	1	48	0.159	43.8	0.912	65	0.797	1.98	25
	12	0.631	41.7	0.1	35.6	0.853	86.9	0.795	1.67	25
	13	0.398	37.2	0.0634	29.2	0.785	119	0.794	1.44	25
	14	0.251	34.6	0.04	24.3	0.703	169	0.791	1.28	25
	15	0.158	35.1	0.0252	21.8	0.619	261	0.792	1.25	25
	16	0.1	36.5	0.0159	20.2	0.555	417	0.789	1.26	25
	f=2.800 Hz, G′=G″=144.2 Pa (cross over), ω(omega)=17.59 rad/s, η (eta)= 4.006.3

**Table:** 

Sample no. 3	Meas. pts.	Angular Frecuency [Rad/s]	Storage Modulus [Pa]	Frecuency [Hz]	Loss Modulus [Pa]	Damping Factor [ 1]	Complex Viscosity [Pa.s]	Deflection Angle [mrad]	Torque [μNm]	Temperature [°C]
**HA hydrogel**	1	100	0	15.9	555	1	5.55	0.8	17	25
	2	63.1	0	10	156	1	2.48	0.801	4.79	25
	3	39.8	79.3	6.34	129	1.62	3.8	0.8	4.63	25
	4	25.1	121	4	120	0.987	6.78	0.801	5.21	25
	5	15.8	121	2.52	105	0.872	10.1	0.801	4.91	25
	6	10	106	1.59	92.3	0.874	14	0.8	4.29	25
	7	6.31	86.2	1	79.5	0.922	18.6	0.799	3.58	25
	8	3.98	67.7	0.634	67	0.99	23.9	0.799	2.91	25
	9	2.51	51.4	0.4	55.4	1.08	30.1	0.8	2.31	25
	10	1.58	38.3	0.252	44.5	1.16	37.1	0.8	1.8	25
	11	1	28.4	0.159	34.9	1.23	45	0.801	1.38	25
	12	0.631	21.3	0.1	26.8	1.26	54.2	0.801	1.05	25
	13	0.398	16.2	0.0634	20.3	1.25	65.2	0.801	0.796	25
	14	0.251	12.8	0.04	15.3	1.2	79.4	0.801	0.611	25
	15	0.158	10.4	0.0252	11.6	1.12	98	0.801	0.476	25
	16	0.1	8.65	0.0159	9	1.04	125	0.8	0.382	25
	f=4.046/0.5988 Hz, G′=G″=119.8/65.48 Pa (cross over), ω(omega)=25.42/3.763 rad/s, η (eta)=236.036 Pa.s									

## Figures and Tables

**Table 1:** Free amino acid contents of HA-QSG hydrogel samples (μg/g).

	Peak name	Retention time (minute)	Area	Concentration μg/g
**4**	L-glycine	15.789	178505	0.015
**5**	L-histidine	16.429	78150	0.008
**6**	L-arginine	20.433	268333	0.038
**9**	L-proline	22.746	87837	0.013
**10**	α-aminobutyric acid	23.960	5936843	N.A
**11**	L-valine	27.038	744300	0.034
**12**	L-methionine	27.583	67347	0.006
**15**	L-isoleucine	30.958	490414	0.020
**16**	**L-leucine**	31.508	1056760	0.049
**17**	**L-phenylalanine**	32.721	415465	0.021

**Table 2:** Particle size statistic parameters of HA-QSG hydrogels: Dv (0.1), Dv (0.5), and Dv (0.9) representing the volume-based percentile diameters, Dv (4.3) indicating the mean volume diameter, and Dv (3.2) indicating the mean surface diameter are reported.

Samples	Dv(0.1)	Dv(0.5)	Dv(0.9)	Dv[3.2]	Dv[4.3]
HA-QSG 1000 μm	148.83 μm	830.37 μm	1499.23 μm	380.23 μm	833.36 μm
HA-QSG 300 μm	83.22 μm	282.59 μm	611.09 μm	183.58 μm	318.22 μm

**Table 3:** Viscoelastic properties of hydrogels for intraarticular injection before and after steam sterilization.

Before thermal sterilization		Viscoelastic properties 0.1 Hz angular frequency		
Sample no.	Samples	G′ [Pa] elastic modulus	G″ [Pa] loss modulus	η* [Pa.s] complex viscosity
1	HA-QSG hydrogel	140.0	63.7	1540.0
2	Free QSG-filled HA hydrogel	51.8	38.2	644.0
3	HA hydrogel	19.8	19.5	278.0
**After thermal sterilization**		**Viscoelastic Properties 0.1 Hz angular frequency**		
**Sample no**.	**Samples**	**G**′ **[Pa] elastic modulus**	**G**″ **[Pa] loss modulus**	η* [Pa.s] complex viscosity
1	HA-QSG hydrogel	92.4	28.2	966.0
2	Free QSG-filled HA hydrogel	66.9	30.3	735.0
3	HA hydrogel	7.33	3.49	81.2

**Table 4:** Viscoelastic properties of hydrogels for dermal filler injection before and after steam sterilization.

Before thermal sterilization		Viscoelastic properties 0.1 Hz angular frequency		
Sample no.	Samples	G′ [Pa] elastic modulus	G″ [Pa] loss modulus	η* [Pa.s] complex viscosity
1	HA-QSG hydrogel	93.6	67.0	1150.0
2	Free QSG-filled HA hydrogel	46.4	44.8	645.0
3	HA hydrogel	17.0	19.7	260.0
**After thermal sterilization**		**Viscoelastic properties 0.1 Hz angular frequency**		
**Sample no**.	**Samples**	**G**′ **[Pa] elastic modulus**	**G**″ **[Pa] loss modulus**	η* [Pa.s] complex viscosity
1	HA-QSG hydrogel	63.0	29.6	696.0
2	Free QSG-filled HA hydrogel	36.5	20.2	417.0
3	HA hydrogel	8.7	9.0	125.0
