# Supplementary figures and images for: Effect of quince seed gum (QSG) on the performance of injectable hyaluronic acid hydrogels in terms of the rheological, morphological, and mechanical aspect
Source: Turk J Chem. 2024 May 29;48(3):422–35. doi: 10.55730/1300-0527.3669 (PMC11265888; doi:10.55730/1300-0527.3669)

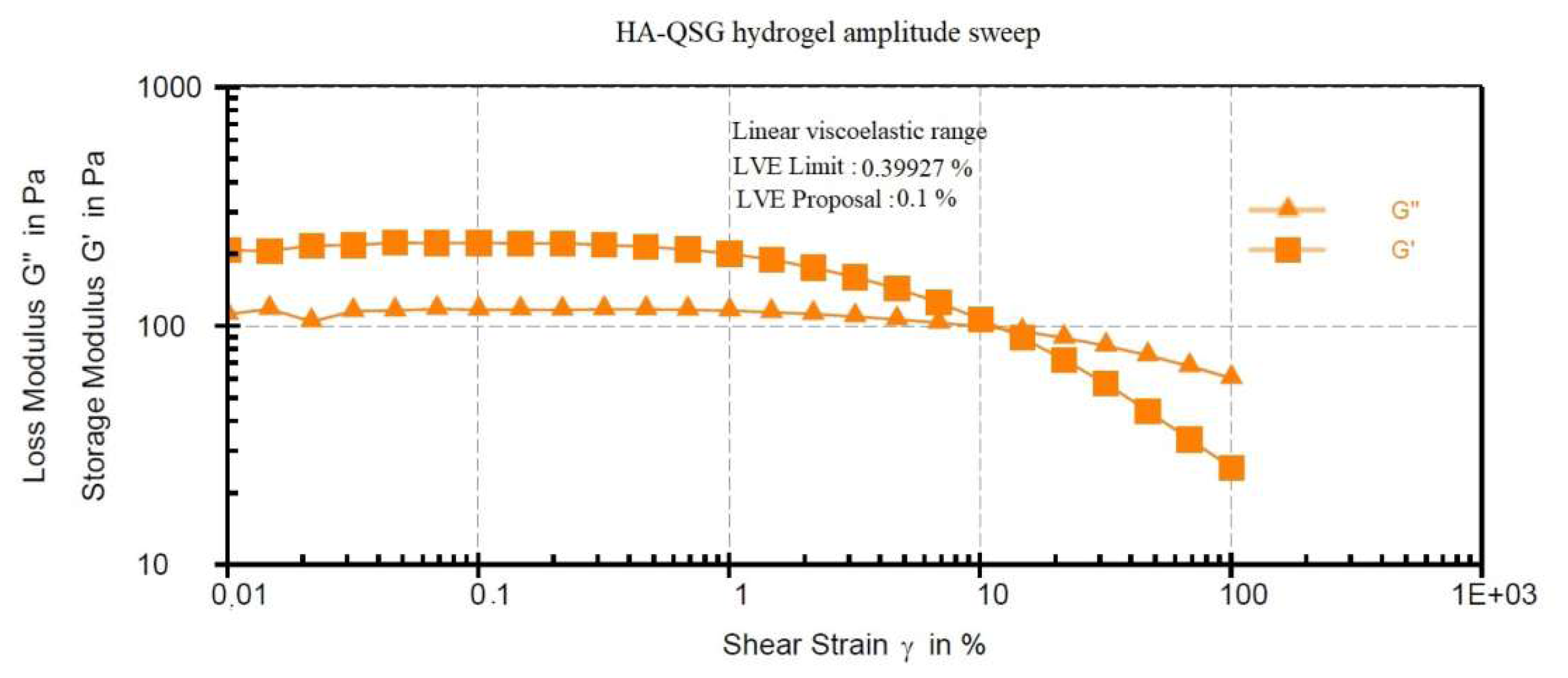

Supplement: Figure S1 — LVE limit graph of HA-QSH hydrogel. [file tjc-48-03-422s1.tif]

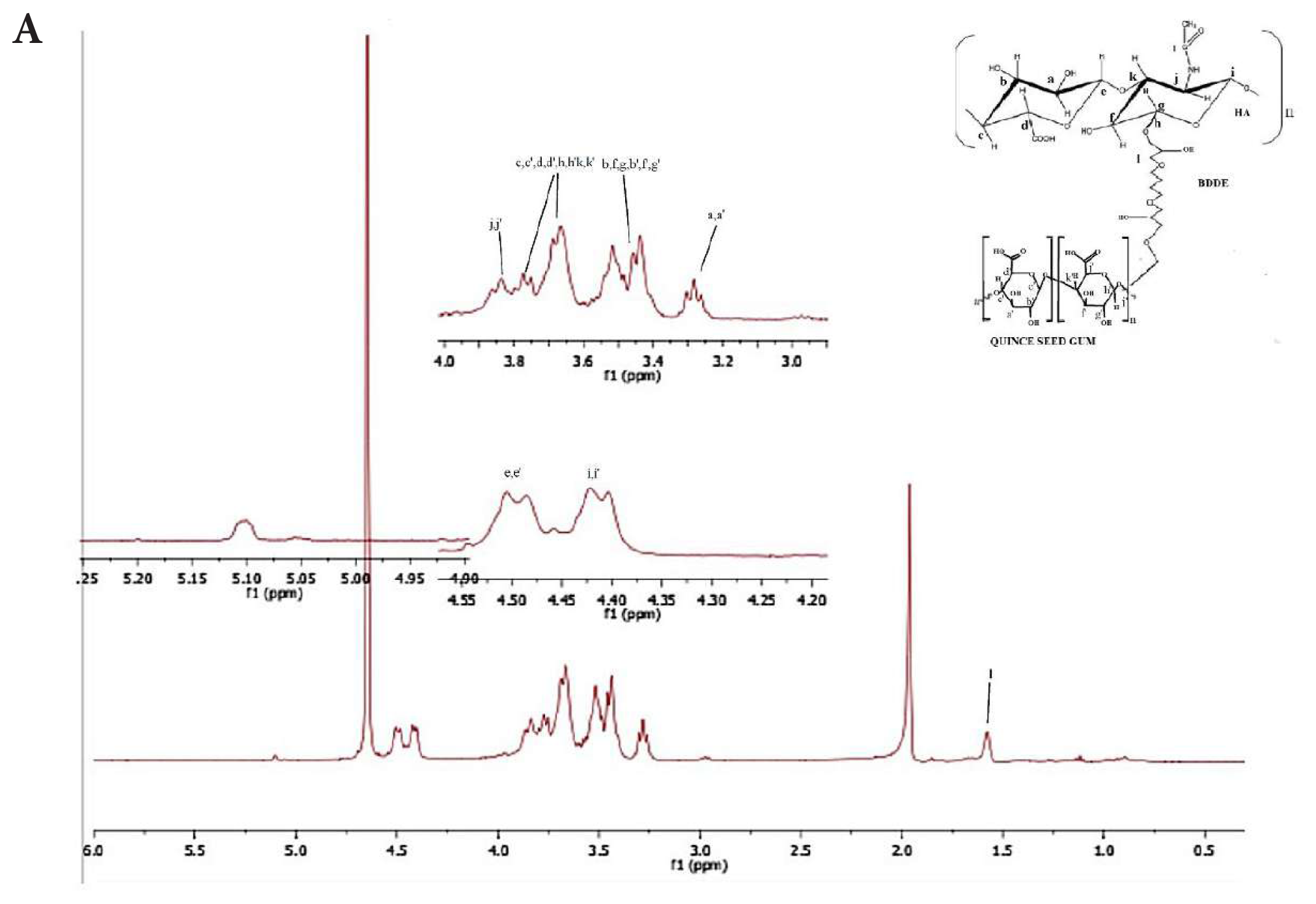

Supplement: Figure S2a — 1H-NMR spectra of HA-QSG hydrogel. [file tjc-48-03-422s2a.tif]

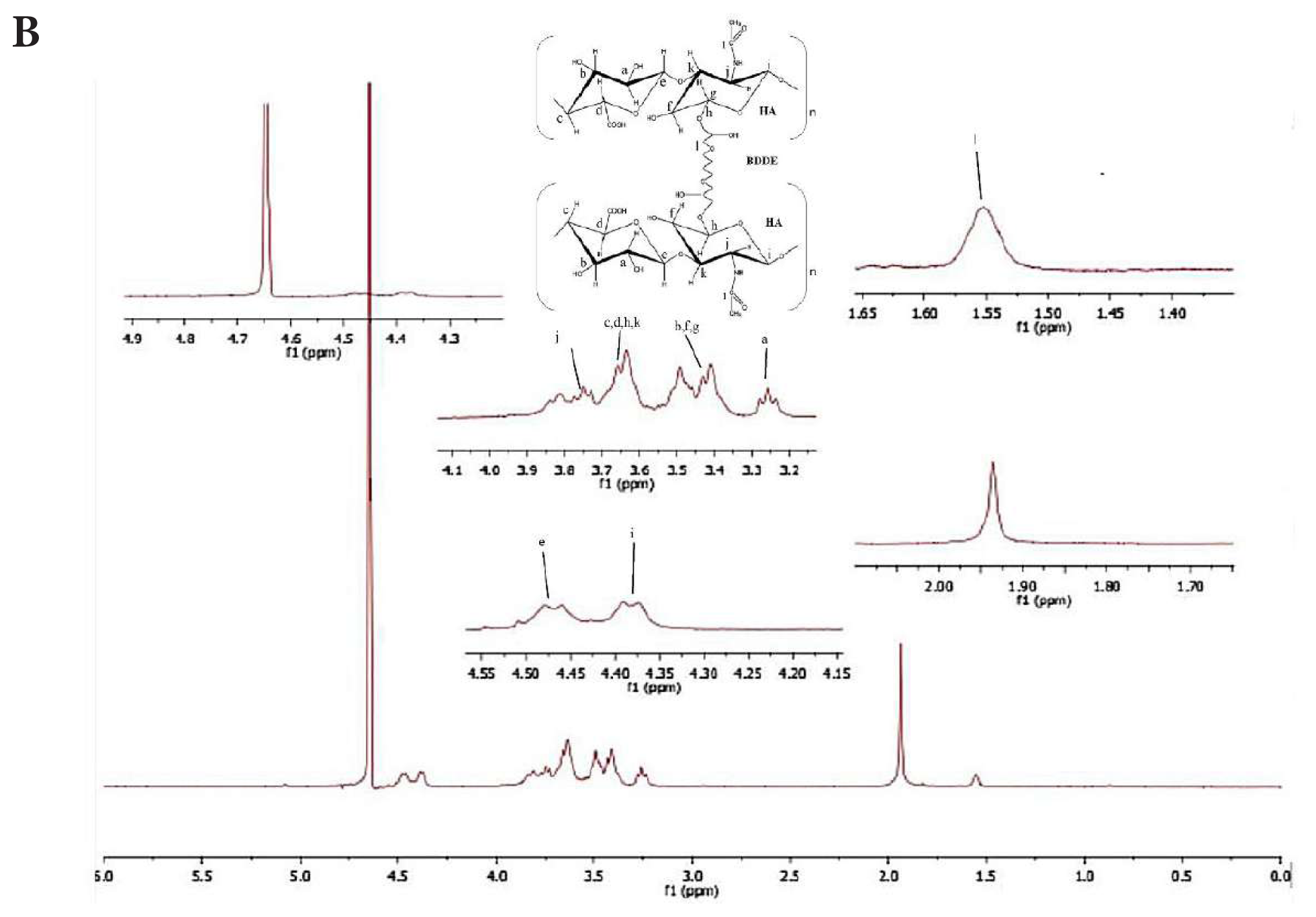

Supplement: Figure S2b — 1H-NMR spectra of HA hydrogel [file tjc-48-03-422s2b.tif]

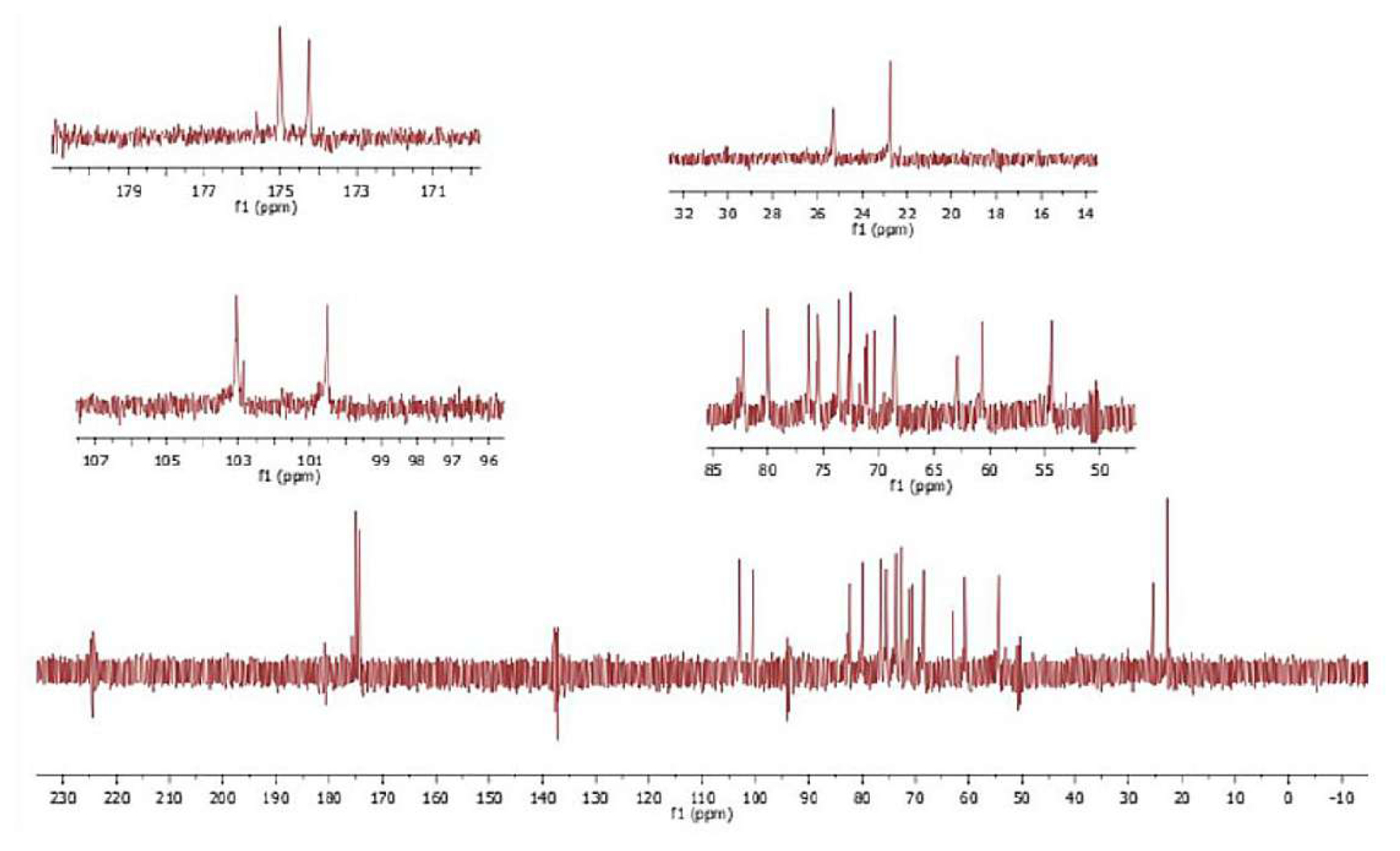

Supplement: Figure S3 — 13C -NMR spectra of HA-QSG hydrogel structure. [file tjc-48-03-422s3.tif]
